# Plant Pathogens Affecting the Establishment of Plant-Symbiont Interaction

**DOI:** 10.3389/fpls.2016.00015

**Published:** 2016-01-21

**Authors:** Eduardo M. de Souza, Camille E. Granada, Raul A. Sperotto

**Affiliations:** ^1^Programa de Pós-Graduação em Biotecnologia, Centro Universitário UNIVATESLajeado, Brazil; ^2^Centro de Gestão Organizacional, Centro Universitário UNIVATESLajeado, Brazil; ^3^Setor de Genética e Biologia Molecular do Museu de Ciências Naturais, Centro de Ciências Biológicas e da Saúde, Centro Universitário UNIVATESLajeado, Brazil

**Keywords:** mycorrhiza, rhizobium, signal transduction, pathogen, symbiont, plant interaction

Hundreds of different microorganisms are attached to the surface of roots. Therefore, it is not surprising that plants have the ability to distinguish threatening intruders from beneficial microbiota (Tóth and Stacey, 2015). Pathogens can be discriminated by plant cells through a myriad of plasma membrane and intracellular receptors that recognize molecules released by microbes, in a process called innate immune system. In spite of the immune ability of plants to prevent pathogen infection, symbiotic signaling molecules are perceived by the host plant, triggering signaling cascades that lead to symbiont infection and accommodation (Tóth and Stacey, 2015). However, some symbiotic signaling molecules can induce responses that are normally associated with plant innate immunity (Pauly et al., 2006), and several observations that are consistent with a rapid, defense-like response occurring in legumes when infected by rhizobia have been obtained, mainly involving programmed cell death, cell wall thickening, reactive oxygen species (ROS) generation, defense phytohormones and salicylic acid (SA) production (Jones and Dangl, 2006; Stacey et al., 2006; Dodds and Rathjen, 2010; Montiel et al., 2012). Similar to bacterial pathogens, symbionts alone also have the ability to actively suppress innate immune response, as previously shown (Liang et al., 2013).

Plant-symbiont-pathogen interaction is an emerging topic, and several questions in this field have been elucidated in the recent years. However, the main focus of these studies is commonly limited to the effects of symbiont microorganisms on the activation of plant defense responses and elicitation of induced systemic resistance to pathogens (Pieterse et al., 2001; de Vleeschauwer and Höfte, 2009), which usually does not come with the normal costs of reduced growth rates and reproductive outcomes in resistance-expressing plants (Spaepen et al., 2009). Studies considering plant pathogens as limiting factors to the symbiosis establishment are still scarce (Faessel et al., 2010; de Román et al., 2011; van Dam and Heil, 2011; Ballhorn et al., 2014). However, such studies are highly relevant for the use of symbiotic inoculum in particular in monocultures of pathogen-susceptible crops. In this opinion article, we focus on rhizobial and mycorrhizal symbiosis inhibition mediated by plant pathogens. We present the current state-of-the-art through the compilation and comparison of available information that can help to elucidate intriguing questions, as the sensing and signaling of plant-symbiont-pathogen interaction.

## Direct antagonism between plant pathogens and symbiont microorganisms

From the moment that plant pathogens and symbionts make plant tissues their major source of space, C, N, and other nutrients, both start to compete frequently. One of the main mechanisms employed in this contest is direct antagonism, which is well studied from the “symbiont against pathogen” perspective (Kumar et al., 2011). This antagonism is based on the production of several antimicrobial products, which act mostly on the rhizosphere (Mucha et al., 2006, 2009; Zou et al., 2007). However, both involved players can attack, defend and counterattack (Raaijmakers et al., 2009). Pathogens are able to respond by inactivating symbiont genes responsible for antimicrobial production, along with changing their own metabolism in order to tolerate the attacks, or even synthesizing substances able to inactivate the antimicrobial product (Duffy et al., 2003; Schouten et al., 2004). Such defense mechanisms against antimicrobial activities initiated by symbionts allow the resistance of soil-borne (on the rhizosphere) and above ground pathogens (on the shoots).

Plant pathogens are also able to attack their opponents using similar molecular weapons (Duffy et al., 2003), which include hydrogen cyanide (Benizri et al., 2005), alkaloids (Antunes et al., 2008), and bacteriocins (Holtsmark et al., 2008). Most of the secondary metabolites produced by plant pathogens are probably unknown, due to the fact that in multi-species communities (as the rhizosphere environment), the myriad of produced compounds tends to be different from that produced in laboratory conditions, using isolated cultures (Netzker et al., 2015). Using such weapons, plant pathogens can modify the structure and abundance of soil microbial populations, including plant symbionts (Mrabet et al., 2006; Liu et al., 2010; Chihaoui et al., 2012), causing deleterious impacts for both plant and microorganism. Even though some studies report a reduction on the symbiosis process in plants inoculated with plant pathogens or even an inhibition on the symbiont development of *in vitro* dual culture, most of them do not investigate the sequential events which lead to symbiosis or symbiont development failure (Muthomi et al., 2007; Lahlali and Hijri, 2010). Such sequential molecular events should be better investigated preferably mimicking natural conditions, in order to be applied to increase the success of agricultural inoculation and plant symbiont interaction.

In a recent report, Sillo et al. (2015) used morphological and gene expression analyses to show an inhibition of the ectomycorrhizal growth caused by phytopathogenic fungus in dual culture conditions. The expression analysis of genes related to cell wall hydrolytic enzymes and hydrophobins, putatively involved in the fungus-fungus interaction, allowed the identification of significantly up- and down-regulated genes in both symbiont and pathogens. Apparently, the inhibition process involves chitinolytic enzymes. As the mRNA levels can be different from the protein levels or activities, the study of both fungal secretomes and metabolomes could be effective for a better understanding of this process.

## Mycorrhizal and rhizobial symbiosis indirectly inhibited by plant pathogens

Plants present several mechanisms to control infections by deleterious organisms. One of the most rapid defense reactions to pathogen attack is the so-called oxidative burst, which includes ROS production (Apel and Hirt, 2004; Gechev et al., 2006; Nanda et al., 2010), along with synthesis of the endogenous signaling molecule salicylic acid (SA—de Román et al., 2011). ROS cause directly strengthening of cell walls via cross-linking of glycoproteins (Delaney et al., 1994; Torres et al., 2006) and SA activates synthesis of chitinase and β-1,3-glucanase, which contribute to a broad-spectrum resistance against diverse bacteria, fungi and viruses (de Román et al., 2011).

Some of the resistance mechanisms, however, may exert ecological costs when they have a negative effect on beneficial plant-microbe interactions. Even though there is increasing evidence that ROS are needed to fully establish the symbiosis, Lohar et al. (2007), Cárdenas et al. (2008) and Munoz et al. (2012) related that ROS elevation might provoke a rhizobial infection abortion in *Medicago truncatula, Phaseolus vulgaris*, and *Glycine max* plants, respectively. Since ROS can act as secondary messengers impacting many processes during plant defense, the elucidation of the mechanisms that control ROS signaling during symbiosis could contribute in defining a powerful strategy to enhance the efficiency of the symbiotic interaction. Also, Blilou et al. (1999) and Stacey et al. (2006) showed that reduced levels of SA results in increased rhizobial infection in *Lotus japonicus, M. truncatula*, and *Pisum sativum*. Exogenous SA application in alfalfa plants results in inhibition of nodule primordia formation and reduction in emerging nodules number (Martínez-Abarca et al., 1998). Interestingly, exogenous SA application completely blocks nodulation of *Vicia sativa* (vetch, an indeterminate-type nodulating plant) by *Rhizobium leguminosarum*. In contrast, addition of SA to *Lotus japonicus* (a determinate-type nodulating plant) does not inhibit nodulation by *Mesorhizobium loti* (van Spronsen et al., 2003). Further efforts should be made to find molecular mechanisms that regulate the different signal transduction pathways of indeterminate- and determinate-type nodulating plants in response to SA.

Mycorrhizal infection is also probably being influenced by SA-dependent defense mechanisms, since enhanced SA levels are detected in mycorrhiza-resistant mutant (*myc*^−^) of *Pisum sativum* in comparison to wild type plants (Blilou et al., 1999), and exogenous SA applied to rice roots reduces mycorrhization at the early stage of plant infection (Blilou et al., 2000). Also, SA reduction leads to elevation of mycorrhizal colonization, infection units, and arbuscules. On the contrary, in tobacco plants that constitutively produce elevated levels of SA, lower colonization levels are observed (Herrera Medina et al., 2003). During rhizobial colonization, SA seems to suppress infection thread formation, but for mycorrhizal colonization the exact stage of inhibition has not been described, although prepenetration apparatus formation seems to be a good target candidate (Gutjahr and Paszkowski, 2009).

Such negative effects may even cross the border between a plant's aerial parts and its roots (de Román et al., 2011; van Dam and Heil, 2011). Induction of SA-dependent resistance to pathogens in foliar tissues of soybean plants, transiently inhibit the mycorrhization of soybean roots (Faessel et al., 2010; de Román et al., 2011), confirming a negative impact of the elicitation of foliar defenses on root-mycorrhizal interactions. According to de Román et al. (2011), the negative effect is likely linked to changes in the defense status of the plant rather than to changes in resource allocation patterns, since no allocation or fitness costs associated with the induction of resistance are detected. Recently, Ballhorn et al. (2014) showed that an aboveground hemibiotrophic plant pathogen induces a defense response that inhibits the belowground mycorrhizal colonization, and that systemically induced polyphenol oxidase activity is functionally involved in this aboveground-belowground interaction.

Induced plant resistance against pathogen causes no significant effect on the frequency of mycorrhizal colonization in soybean roots, but reduces the intensity of colonization and the proportion of arbuscules, along with the number of *Bradyrhizobium* nodules (Faessel et al., 2010). A similar pattern was shown in pea *myc*^−2^ mutants (Gianinazzi-Pearson et al., 1991), in which the fungus is able to form appressoria and penetrate into roots, but fails to form arbuscules. Also, the mutant *myc*^−2^ was characterized by an enhanced expression of the *Psam4* (*Pisum sativum arbuscular mycorrhiza-regulated*) gene, which encodes a proline-rich protein, generally associated with cell wall strengthening in plant-pathogen interaction (Marsh and Schultze, 2001). In *Medicago truncatula* hairy roots, PR-10 (pathogenesis-related-10) protein is upregulated in root cells close to the hyphopodium and subsequently repressed during formation and fungal passage of the prepenetration apparatus (Siciliano et al., 2007). Only local, weak, and transient defense responses are activated during early steps of beneficial plant microbe interactions, and low amounts of ROS and SA are necessary in the earlier steps of both rhizobial and mycorrhyzal symbiosis (Faessel et al., 2010), facilitating the access of these microorganisms inside the plant tissue. Local defense responses, such as chitinase and β-1,3-glucanase activities are enhanced during early steps of compatible mycorrhizal interactions (Pozo and Azcón-Aguilar, 2007), but these enzymatic activities are repressed at a later stage of mycorrhiza formation. Rhizobia produce cyclic β-glucans suppressing the induction of plant defense (Mithöfer et al., 1996), starting cortical cells division which lead to a nodule primordium (Tóth and Stacey, 2015). It would be interesting to test whether the spatio-temporal expression pattern of PR-10 protein correlates with SA activity, as well as combining SA application with high-resolution imaging in order to check the effect of SA in early infection structures.

It is important to highlight that some studies use acibenzolar S-methyl (ASM) as a synthetic inducer of the SA pathway. ASM induces successful disease resistance in many plant-pathogen combinations, but there are also reports where it did not significantly induce resistance (Heil, 2007). Therefore, effects of ASM have to be evaluated for each plant-pathogen combination. Unexpectedly, the P content of soybean increases when ASM is applied to leaves (Faessel et al., 2010). One plausible explanation is that ASM interacts with root exudation of organic acids, which in turn mobilize insoluble P. Another explanation would be that ASM regulates still unknown functions of plant development which compensate mycorrhization inhibition, probably caused by enhanced P nutrition. Field studies are required to confirm these side-effects and their possible consequences on crop yield. This is an example why experiments should preferably be carried out at densities and scenarios as realistic as possible, since we can expect plants to be adapted only to events that are common over evolutionary time spans. Future studies aiming toward the identification of the mechanisms in this co-operative effort should involve studying the interaction under different nutrient conditions and densities of rhizobial/mycorrhizal colonization and monitoring physiological and transcriptomic changes in both plant roots and symbiont microorganism during the various phases of the interaction.

## Future perspectives

We must admit that there is a gap on the evaluation of plant pathogen effects over the symbiosis capacity of rhizobia and mycorrhiza. This type of analysis should be more and more stimulated, since the evaluation of putative interferences from plant pathogens in plant-symbiont interactions is mandatory to enhance the effectiveness of the application of agricultural inoculants in natural environments. It is already known that pathogens and symbionts might utilize identical or overlapping molecular mechanisms for their colonization (Wang et al., 2012; Rey et al., 2015), further complicating the study of plant-symbiont-pathogen interaction. In order to better understand this triple interaction, *omic* analyses in controlled systems, containing the host plant and micro-symbionts, could provide valuable information to complement knowledge acquired through studies on the competition of pathogen and symbiont. Such studies can identify molecular players which act in different levels (RNAs, proteins, enzymatic activities, metabolites, inhibitors, and enhancers), and some of them are probably putative targets for enhanced efficiency of plant-symbiont interaction.

## Author contributions

ED, CG, and RS wrote the paper.

### Conflict of interest statement

The authors declare that the research was conducted in the absence of any commercial or financial relationships that could be construed as a potential conflict of interest. The Guest Associate Editor Ralph Panstruga and Reviewer Hannah Kuhn declared their shared affiliation, and the Editor states that the process nevertheless met the standards of a fair and objective review.
